# Virtual Reality for Chronic Pain Management Among Historically Marginalized Populations: Systematic Review of Usability Studies

**DOI:** 10.2196/40044

**Published:** 2023-06-06

**Authors:** Marika Dy, Kristan Olazo, Sarah Lisker, Ellenor Brown, Anindita Saha, Jessica Weinberg, Urmimala Sarkar

**Affiliations:** 1 Department of Medicine Division of General Internal Medicine University of California San Francisco San Francisco, CA United States; 2 Center for Vulnerable Populations Zuckerberg San Francisco General Hospital and Trauma Center University of California San Francisco San Francisco, CA United States; 3 Center for Devices and Radiological Health US Food and Drug Administration White Oak, MD United States

**Keywords:** virtual reality, chronic pain management, systematic review, historically marginalized populations, VR, usability, pain management, pain, chronic pain

## Abstract

**Background:**

Virtual reality (VR) has potential to improve chronic pain management outcomes. However, the majority of studies assessing VR are conducted in predominantly White populations in well-resourced settings, thus leaving a gap in knowledge of VR use among diverse populations who experience a significant chronic pain burden.

**Objective:**

This review aims to examine the extent to which usability of VR for chronic pain management has been studied within historically marginalized patient groups.

**Methods:**

We conducted a systematic search to identify studies with usability outcomes located in high-income countries that included a historically marginalized population, defined by a mean age greater than or equal to 65 years, lower educational attainment (greater than or equal to 60% having attained high school education or less), and being a racial or ethnic minority (less than or equal to 50% non-Hispanic White people for studies based in the United States).

**Results:**

Our analysis included 5 papers, which we used to conduct a narrative analysis. Three studies examined VR usability as a primary outcome. All studies assessed VR usability using different measures, of which 4 found VR to be usable by their respective study population. Only 1 study found a significant improvement in pain levels post–VR intervention.

**Conclusions:**

The use of VR shows promise for chronic pain management, but few studies include populations that are older, have limited educational attainment, or have racial or ethnic diversity. Additional studies with these populations are needed to further develop VR systems that work best for diverse patients with chronic pain.

## Introduction

### Background

Virtual reality (VR) has been defined as the use of computer technology to create the effect of an interactive 3D world in which the objects have a sense of spatial presence [1]. Over the past 2 decades, VR has been tested and used in a variety of health care settings, including medical education or training, physical rehabilitation, pain management and distraction from procedure-related symptoms (eg, anxiety and distress), and treatment of psychiatric disorders (eg, phobias) [2-9].

### VR Capabilities for Chronic Pain

There is evidence on the immediate effectiveness of VR on acute and chronic pain management [10-13]. In clinical settings, patients using VR have exhibited reduced levels of pain, anxiety, and distress during medical procedures and experimental stimuli [14]. Although exact neuropsychological mechanisms behind VR’s effect are unclear, it is thought that VR may influence the body’s pain signaling pathway [15]. To be effective for chronic pain, VR treatments must be feasible for patients to navigate and use, particularly because patients with chronic pain play a more active role in their pain management, self-administering medications, and engaging with nonpharmacological approaches compared to patients with acute pain.

### Disparities in Chronic Pain Treatment

Because there are significant disparities in chronic pain incidence and treatment [16-18], it is critical to examine the usability of VR among these groups in order to determine whether VR as chronic pain treatment can be widely used. Among older adults, chronic pain is highly prevalent and is associated with greater health costs. However, older adults are limited in their ability to take medication due to side effects that may result in additional health risks, such as cognitive impairment or increased risk of falls [19]. Additionally, in the United States, those with a high school education or less have been found to be more likely to experience severe chronic pain in comparison to college graduates [20]. Moreover, Black patients report significantly increased pain severity in comparison to White patients [21-26]. Despite facing an unequal burden of pain, Black patients are 22% less likely than White patients to receive any analgesics as a result of physician perceptions, bias, and racism [27]. Uninsured patients, as well as those from low socioeconomic backgrounds or minority groups, are also more likely to experience delays in care and poorly coordinated care and are less likely to have access to primary care [28], where most chronic pain is managed in the United States [29]. Even if patients are able to access and receive chronic pain treatment, it can lead to problematic and potentially fatal opioid usage if not prescribed and monitored carefully [30]. Decisions about pain management are complex, particularly for chronic conditions that have no physical indicators of disease (eg, complex regional pain syndrome and fibromyalgia) [16], leading to physician reliance on stereotypes when under conditions of uncertainty [31,32].

### Opportunities for VR to Address Pain Disparities

With a clear divide in medication treatment for historically marginalized patients who need it most, along with the risks of opioids [33,34], there is an urgent need for alternative options in chronic pain management. VR and other wearable devices act as an adjunct treatment option for acute and chronic pain. In previous studies, patient beliefs that medication use is the best or an important treatment method decreased over time after completing alternative pain management programs (ie, meditation, physical therapy, and cognitive behavioral therapy) [35,36]. Patients have demonstrated interest in using technology to manage their health [37,38] as well as clinician interest to use VR as an adjunct or replacement to opioid agents [39]. With benefits of safety, portability, and potentially affordable relative costs, VR may be an ideal option for patient groups who are unable to access care and other treatment methods. However, even with evidence proving the efficacy and feasibility of VR therapies for pain management, standard care for chronic pain has yet to integrate VR as part of it due to implementation barriers such as cost, need to dedicate additional time to learn how to use the VR device, and lack of culturally relevant VR content [39-41]. Understanding more about its usability and efficacy will help apply this method of treatment to diverse patients who could benefit from this technology.

To determine usability, device designers should assess the quality of a user’s experience as they interact with the product [42]. By gathering information on device usability, using user feedback can improve these tools to be appropriately and effectively designed for end users of the intervention [43]. In existing literature, there is an evaluation gap on the use of VR therapy among populations that have been historically marginalized. This poses a barrier to clinical uptake in safety-net settings, which provide health care and other services for low-income and racially or ethnically diverse patients who are on Medicaid or are uninsured [44]. In the United States, safety-net health systems rely on local, state, and federal funding to provide care to populations who are uninsured or underinsured and therefore, would otherwise lack access to care [45]. Safety-net health systems stand to benefit from innovations like VR, as they often lack access to scalable, effective technology interventions and are at the front line of care for patients at risk of experiencing health and health care disparities [46]. Even when available, such tools may not be successfully implemented in safety-net settings if they were not also designed for historically marginalized patients as the intended users [47,48]. It is clear that VR, as well as other digital tools, must be designed differently from those that are used in and designed for White populations so that it is usable and relevant for diverse populations.

In a previous study, health care providers and leaders from safety-net settings identified VR to be useful, scalable, and an appealing alternative to existing pain management approaches [39]. Current providers using VR noted positive patient feedback but raised concerns that existing VR content may not be engaging for diverse patients and may pose challenges for patients who are not comfortable using technology [39]. Thus, this literature review will show that there is very little current research that has explored VR usability for historically marginalized patient groups. This encompassed understanding the studied patient populations, the usability of VR therapy headsets, and the relevancy of VR content shown.

## Methods

This review largely followed Preferred Reporting Items for Systematic Reviews and Meta-Analyses (PRISMA) guidelines [49], though there is no statistical analysis, as this is a systematic literature review and not a meta-analysis.

### Search Strategy

We performed a search of the literature in PubMed on May 20, 2021. The search strategy combined 2 main concepts: VR for pain management and usability testing, including search terms reflecting feasibility and acceptability (Table 1).

We applied Boolean logic to the search by combining similar terms with OR and using AND between the 2 concepts; examples include “virtual reality” [Mesh] AND (“pain” OR “analgesic”) [tiab]. Due to the specific focus of our review on VR for chronic pain, this search was conducted only within the biomedical literature in PubMed with no date restrictions.

**Table 1:** Search strategy details.

Date	Database searched	Search strategy	Results, n
May 20, 2021	PubMed (2000-present)	(((“VR”[Mesh]) OR (“virtual reality”[Mesh])) AND ((“pain”[tiab) OR (“analgesia”[tiab])) AND ((“management”[tiab]) OR (“distraction”[tiab])) [232] AND (((“VR”[Mesh]) OR (“virtual reality”[Mesh])) AND ((“pain”[tiab]) OR (“analgesia”[tiab])) AND ((“usability”[tiab]) OR (“feasibility”[tiab]) OR (“acceptability”[tiab]))) [63]	295

### Inclusion Criteria

This review focuses on studies that included at least one historically marginalized population, which we define using the following criteria: mean age (≥65 years), education (≥60% high school education or less), or race or ethnicity (≤50% non-Hispanic White for US-based studies; race or ethnicity standards do not apply to non-US studies). We have chosen to use these 3 identities because all 3 are groups that face chronic pain disparities [19,20,50,51]. Our rationale to include study populations with a mean age of ≥65 years follows the World Bank definition of older adults [52]. We included studies with ≥60% high school education or less to ensure that studies included a majority of adults with low educational attainment given that 66% of the world has attained a secondary education [53]. Studies with ≤50% non-Hispanic White people were also included for US-based studies to allow for equitable representation of patients of color who are frequently not included in clinical research [54]. Additionally, we focused on studies in high-income countries, as defined by the World Bank [55], due to relatively matched rates of technology uptake and advancement [56]. Further, we included papers that examined VR usability for chronic pain management, which we define as studies that evaluate “how effectively, efficiently and satisfactorily users” with chronic pain could interact with VR [57].

### Exclusion Criteria

Papers were reviewed and excluded at 2 levels. At the first screening level, we reviewed titles and abstracts and excluded papers if they were not original research, were single case reports, or studied VR usability to manage experimental pain, acute pain, procedure-related pain, or psychological symptoms (eg, anxiety, depression). These studies were excluded because the interventions would potentially involve strategies specific to a population group that does not experience physical chronic pain, thus making it difficult to generalize their application to patients with chronic pain. Any pediatric studies were excluded due to interviews and surveys focusing on providers and guardians of patients, rather than the patient’s own input.

At the second level, the full text of the papers was reviewed. We excluded papers that we could not retrieve full text of, did not collect participant demographics, did not assess VR usability, or were not conducted in high-income countries. Papers that did not include at least one historically marginalized patient group were also excluded; this specifically excluded populations that were <60% high school educated or less, were aged <65 years, or >50% non-Hispanic White for US-based studies. Due to lower- to upper middle–income countries facing barriers to technology advancement [56], we excluded studies conducted in these countries to account for device feature differences that may limit VR usability.

### Data Analysis

A spreadsheet was developed in Excel (Microsoft Corp) to extract study characteristics. Data extracted from included studies were study design, sample size, study setting, participant demographics, outcome measures, measurement tools, and study findings. MD completed title and abstract screening, as well as full-text screening. Given that the data we aimed to extract are fixed and leave no room for interpretation, we were able to ensure uniform data extraction and analysis.

## Results

### Overview

**Figure 1:**
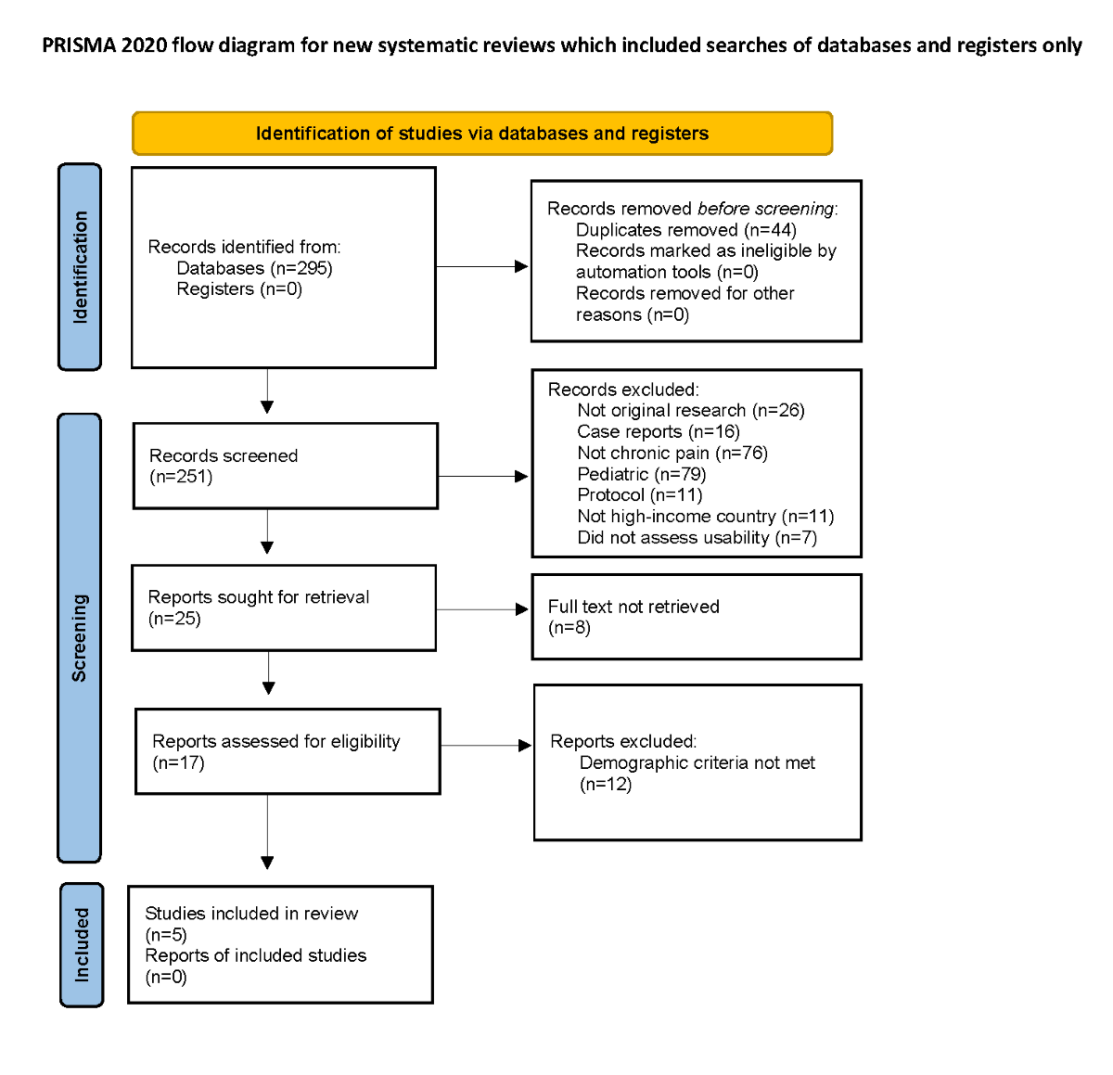
PRISMA (Preferred Reporting Items for Systematic Reviews and Meta-Analyses) flow diagram.

In total, 295 studies were identified using our search criteria, of which 44 duplicates were removed (Figure 1). We then screened 251 papers for inclusion based on title and abstract, of which we excluded 226 papers based on exclusion criteria. Of the remaining 17 studies, 12 were excluded because they did not meet demographic criteria (Multimedia Appendix 1). Overall, 4 studies were missing the necessary data, and 8 studies did not have a sufficient number of participants from a historically marginalized group. Thus, only 5 studies were found that evaluated usability of VR for chronic pain management among populations that have been historically marginalized (Table 2). All included studies collected 1-3 participant demographic characteristics and were published in the past 6 years (2015-2020).

Of these studies, 3 were conducted in the United States [58-60], 1 was conducted in Spain [61], and 1 was conducted in Germany [62]. Across all 5 studies, participants rated VR very favorably as a pain management strategy.

**Table 2:** Study characteristics (n=5).

Author (year of publication)	Study design	Size, N	Population demographics	Study objective	Usability testing method	Usability outcome	Findings
Benham et al (2019) [59]	Observational (mixed methods exploratory)	12	92% White 67% female Mean age 70.2 (SD 3.6) years^a^	Examine applicability and effectiveness of a VR^b^ intervention for pain, depression, and quality of life in older adults.	Qualitative interviews	Qualitative themes: usability assessment and satisfaction	All participants reported overall positive experiences. Overall, 47% experienced an undesirable symptom, but all symptoms were related to a specific activity that could be avoided in future use; 91.7% reported they would continue use if given the chance; and 100% would recommend the device to other seniors.
Fowler et al (2019) [58]	Observational (single-arm feasibility)	16	50% White, 25% African American or Black, 12% Hispanic or Latino, and 13% other^a^ Mean age 48.88 (11.62) years 81% male	Assess effect and feasibility of VR usage on fear of movement, pain outcomes, and patient functioning.	Quantitative questionnaire	Ratings of immersion, self-reported VR intensity, session length, and side effects	Selected applications did not calibrate in intensity as expected (low-movement applications rated higher in intensity and high-movement applications rated lower). Sessions were also rated too short. Minor adverse events included cybersickness symptoms and neck strain.
Garcia-Palacios et al (2015) [61]	Randomized control trial	61	100% White 100% female Mean age 50.48 (SD 9.78) years 21% less than elementary level of education, 36% elementary level of education, 31% completed high school, and 11% completed university^a^ 79% married, 10% single, 10% divorced, and 2% widow	Assess acceptability and efficacy of VR based on activity level, pain, quality of life, and mood of patients with fibromyalgia.	Quantitative questionnaire	Rating of acceptability and satisfaction	Participants had high satisfaction with VR treatment. They found the treatment logical and useful and would definitely recommend it to a friend. The participants did not find the treatment aversive or unpleasant. The VR device was deemed highly useful.
Hennessy et al (2020) [60]	Observational (content validity and feasibility)	12	100% Black^a^ 67% female Mean age 54.3 (SD 5.1) years	Determine content validity and feasibility of VR application use.	Usability questionnaire (SUS^c^)	Ratings of content validity; SUS	All participants assigned higher avoidance, expected pain, and expected concern for high-intensity modules compared with low-intensity modules. Participants rated the usability of the VR application as acceptable.
Stamm et al (2020) [62]	Observational (usability evaluation)	15	Mean age 75.9 (SD 6.9) yearsa	Understand expectations, desires, preferences, and barriers of VR pain therapy. Determine frameworks of therapy by physiotherapists and psychotherapists.	Qualitative questionnaire	Expert and user requirements assessments	Key requirements were target-group–specific and included individual briefing, user-friendly handling, inclusion of movement limitations, presentation of everyday scenarios in combination with biofeedback, age-appropriate feedback through praise and awards, and a maximum exercise duration of 30 and 15 min of relaxation.

^a^Reported study demographic that met inclusion criteria.

^b^VR: virtual reality.

^c^SUS: System Usability Scale.

### Age

Stamm et al [62] and Benham et al [59] specifically sought to examine usability among adults aged 65 years or older. Age was the only demographic reported by the former study, whereas the latter also reported gender and race, which revealed study participants to be majorly female and White. Additionally, Stamm et al [62] focused on testing VR with participants experiencing chronic back pain, and while VR usability as an outcome was an inclusion criterion met by all included studies, their study had usability as a primary study outcome, and Benham et al [59] had it as a secondary outcome. Both studies deployed qualitative questionnaires and interviews, respectively. However, only Benham et al [59] asked participants about their overall experience with VR in addition to exploring the usability of the device. All participants found VR to be a positive experience and would recommend the device. However, Benham et al [59] also found that almost half of the study’s participants experienced an undesirable symptom that was related to a VR activity that could be avoided in the future.

Stamm et al [62] solely conducted a requirement analysis of the VR device; thus, they did not inquire about patient satisfaction with the device. Instead, this study found key elements and content that should be included in VR to make it relevant and usable for patients and health care providers, including device tutorials, user-friendly handling, and positive system feedback. Both studies explicitly recommended supervision of VR usage if applying the device to groups who face difficulties navigating the device (ie, patients with low digital literacy) and when used by older adults due to the possibility of adverse side effects from VR.

### Education

Garcia-Palacios et al [61] was the only study we found that included a significant percentage of participants who were high school educated or less. This study was conducted in Spain, and the majority of study participants were female; the study authors did not mention intentional recruitment of participants who were high school educated or less. They had VR usability as a secondary outcome. To assess usability, they used an adapted quantitative scale to assess participant satisfaction and acceptability with the VR treatment. Patients found the VR system logical and useful and rated the overall treatment positively. This study did not gather data to evaluate VR content relevancy.

### Race

Hennessy et al [60] and Fowler et al [58] did not mention intentional recruitment of participants from racial or ethnic minority backgrounds; however, the former study specifically focused on evaluating VR with participants experiencing chronic back pain. Both studies examined VR usability as a primary study outcome. Hennessy et al [60] conducted a feasibility trial to determine content validity of a VR-graded exposure approach for patients with chronic low back pain. Participants deemed the VR treatment usable through the System Usability Scale [63]. This was the only study to use the System Usability Scale as a primary measure of intervention usability. Fowler et al [58] assessed usability through a quantitative rating questionnaire. Even though it was not significant, participants reported minor side effects and heaviness of the VR headset. In general, participants rated 20-minute VR sessions as too short. Finally, both studies found that participants expressed concern about using VR because some VR content required higher levels of activity intensity than manageable for them.

## Discussion

### Principal Findings

By examining existing literature on VR usability for chronic pain management, our findings expand beyond prior systematic reviews that found VR to be effective [64-66]. To our knowledge, there are no other reviews on the usability of VR for chronic pain management. By specifically including studies that included historically marginalized participants, our results offer the first comprehensive assessment of existing literature on VR usability for chronic pain management among marginalized populations, who are most likely to experience high burdens of disease and health inequities. Although we found 5 studies that met our review’s parameters, our findings point out a clear need for future studies in VR for pain management to intentionally include historically marginalized groups who frequently do not have access to alternative pain management strategies [67,68].

Even if these population groups are offered VR, the technology is currently designed to cater toward usability and content relevancy needs of White patients with higher income. In particular, we only found 2 studies that included a significant percentage of racially marginalized patients [58,60], despite their lower likelihood to be prescribed analgesics or more likely to be given lower dosages compared to White patients [16,27,69]. Additionally, the identities of historically marginalized populations do not exist in isolation and are intersectional. For example, Black and Hispanic patients are more likely to have a high school education or less than White students [70]. Should VR devices be designed to target historically marginalized populations, there would still remain a wide range of experiences and user needs. This requires deeper research that focuses on understanding usability and content relevancy needs of diverse patient groups.

Furthermore, we found 4 studies that examined VR content relevancy within their respective study populations [58-60,62]. However, these study results revealed a need for VR modules that were adjusted to the limited movement capabilities of patients with chronic pain in order to avoid overexertion or kinesiophobia. We did not find any relevancy results in relation to cultural and linguistic needs to improve VR usability for diverse patients. Expanding innovations such as VR to be culturally relevant and usable for historically marginalized patients living with chronic pain is an unrealized opportunity to improve their quality of life.

Moreover, although the 5 studies included historically marginalized patients, only 2 studies stated their intentional inclusion of these groups [59,62]. As such, the other 3 studies did not explicitly consider any usability findings to be associated with their respective study population. This makes it difficult to conclude that their findings will be applicable to the same type of patient groups. To address this clinical need and fill this gap in knowledge, we are conducting a study that reveals the opportunity for culturally relevant and usable VR for diverse safety-net patients experiencing chronic pain. To our knowledge, our study examining the usability of VR for pain management will be the first conducted in a safety-net setting. This will not only add to the sparse number of studies with racially or ethnically diverse, lower-income participants but will help improve our understanding of the technological interface and hardware needs for historically marginalized communities.

### Limitations

This study has several limitations. Due to the wide variation in definitions, measurements, and reporting of outcomes, we used terms reflecting VR, usability testing, and chronic pain in our search strategy to capture studies evaluating VR usability for chronic pain management. It is possible that we have not captured all relevant studies, especially if they used different terminology. Given our specific focus on VR used to manage chronic pain, our search was limited to PubMed as it is the primary database for biomedical literature. Thus, we may have only missed studies available on other databases. Additionally, only 5 studies met the criteria of including a historically marginalized population. Of these studies, only 1 was a randomized control trial, and all had small sample sizes, thereby, limiting the generalizability of our findings. Finally, the studies we included used different measures of usability and pain outcomes. Taken together, this suggests a need to report usability and pain outcomes in a consistent manner in order to facilitate comparisons across studies.

### Conclusions

Despite the limitations of this review, we believe this paper adds to the literature on VR by focusing on the usability of the tool for chronic pain management in historically marginalized populations that face large health disparities. We found VR to be a promising tool for use in these populations as a potential pain treatment alternative, yet this review also highlights the need to specifically include diverse populations and collect sociodemographic data in digital health studies. Given the varying measures of usability, it is important that researchers assessing VR use the same evaluation tools in order to facilitate comparisons for generalizability. Further, digital health technology designers and researchers should consider incorporating strategies, such as co-design or human-centered design, to ensure diverse patient satisfaction [71,72]. This intentional engagement of end users throughout the design process has already been exhibited to help develop digital health care technology that meets the needs and preferences of historically marginalized groups [73-76]. It is critical to ensure VR technologies are usable and relevant for diverse populations. Not only will it help improve patient uptake and sustained technology use [77], but it can also serve as an opportunity to identify any disparities in chronic pain care [78] that may be perpetuated by the growing availability of VR tools, which are heavily studied among and for White users [59,61,79-81].

## Appendix

Multimedia Appendix 1 Characteristics of full texts reviewed.

## Abbreviations

PRISMA: Preferred Reporting Items for Systematic Reviews and Meta-Analyses
VR: virtual reality

## References

[1] Virtual reality: definition and requirements. NASA Advanced Supercomputing Division.

[2] Schneider SM, Ellis M, Coombs WT, Shonkwiler EL, Folsom LC (2003). Virtual reality intervention for older women with breast cancer. Cyberpsychol Behav.

[3] Jack D, Boian R, Merians AS, Tremaine M, Burdea GC, Adamovich SV, Recce M, Poizner H (2001). Virtual reality-enhanced stroke rehabilitation. IEEE Trans Neural Syst Rehabil Eng.

[4] Rothbaum BO, Hodges LF, Kooper R, Opdyke D, Williford JS, North M (1995). Effectiveness of computer-generated (virtual reality) graded exposure in the treatment of acrophobia. Am J Psychiatry.

[5] Hoffman HG, Patterson DR, Carrougher GJ (2000). Use of virtual reality for adjunctive treatment of adult burn pain during physical therapy: a controlled study. Clin J Pain.

[6] Capitani P, Zampogna B, Monaco E, Frizziero A, Moretti L, Losco M, Papalia R (2023). The role of virtual reality in knee arthroscopic simulation: a systematic review. Musculoskelet Surg.

[7] Choi J, Thompson CE, Choi J, Waddill CB, Choi S (2022). Effectiveness of immersive virtual reality in nursing education: systematic review. Nurse Educ.

[8] Barteit S, Lanfermann L, Bärnighausen T, Neuhann F, Beiersmann C (2021). Augmented, mixed, and virtual reality-based head-mounted devices for medical education: systematic review. JMIR Serious Games.

[9] Roswell RO, Cogburn CD, Tocco J, Martinez J, Bangeranye C, Bailenson JN, Wright M, Mieres JH, Smith L (2020). Cultivating empathy through virtual reality: advancing conversations about racism, inequity, and climate in medicine. Acad Med.

[10] Spiegel B, Fuller G, Lopez M, Dupuy T, Noah B, Howard A, Albert M, Tashjian V, Lam R, Ahn J, Dailey F, Rosen BT, Vrahas M, Little M, Garlich J, Dzubur E, IsHak W, Danovitch I (2019). Virtual reality for management of pain in hospitalized patients: a randomized comparative effectiveness trial. PLoS One.

[11] Bani Mohammad E, Ahmad M (2019). Virtual reality as a distraction technique for pain and anxiety among patients with breast cancer: a randomized control trial. Palliat Support Care.

[12] Matheve T, Bogaerts K, Timmermans A (2020). Virtual reality distraction induces hypoalgesia in patients with chronic low back pain: a randomized controlled trial. J Neuroeng Rehabil.

[13] Carrougher GJ, Hoffman HG, Nakamura D, Lezotte D, Soltani M, Leahy L, Engrav LH, Patterson DR (2009). The effect of virtual reality on pain and range of motion in adults with burn injuries. J Burn Care Res.

[14] Li A, Montaño Z, Chen VJ, Gold JI (2011). Virtual reality and pain management: current trends and future directions. Pain Manag.

[15] Abiodun AO, Adesina MA (2019). Virtual reality: a breakthrough in pain management?. World News Nat Sciences.

[16] Green CR, Anderson KO, Baker TA, Campbell LC, Decker S, Fillingim RB, Kalauokalani DA, Lasch KE, Myers C, Tait RC, Todd KH, Vallerand AH (2003). The unequal burden of pain: confronting racial and ethnic disparities in pain. Pain Med.

[17] Dahlhamer J, Lucas J, Zelaya C, Nahin R, Mackey S, DeBar L, Kerns R, Von Korff M, Porter L, Helmick C (2018). Prevalence of chronic pain and high-impact chronic pain among adults—United States, 2016. MMWR Morb Mortal Wkly Rep.

[18] Anderson KO, Green CR, Payne R (2009). Racial and ethnic disparities in pain: causes and consequences of unequal care. J Pain.

[19] Domenichiello AF, Ramsden CE (2019). The silent epidemic of chronic pain in older adults. Prog Neuropsychopharmacol Biol Psychiatry.

[20] Grol-Prokopczyk H (2017). Sociodemographic disparities in chronic pain, based on 12-year longitudinal data. Pain.

[21] Zajacova A, Grol-Prokopczyk H, Zimmer Z (2021). Pain trends among American adults, 2002-2018: patterns, disparities, and correlates. Demography.

[22] Green CR, Baker TA, Sato Y, Washington TL, Smith EM (2003). Race and chronic pain: a comparative study of young black and white Americans presenting for management. J Pain.

[23] Haq N, McMahan VM, Torres A, Santos G-M, Knight K, Kushel M, Coffin PO (2021). Race, pain, and opioids among patients with chronic pain in a safety-net health system. Drug Alcohol Depend.

[24] Edwards RR, Doleys DM, Fillingim RB, Lowery D (2001). Ethnic differences in pain tolerance: clinical implications in a chronic pain population. Psychosom Med.

[25] Riley JL, Wade JB, Myers CD, Sheffield D, Papas RK, Price DD (2002). Racial/ethnic differences in the experience of chronic pain. Pain.

[26] McCracken LM, Matthews AK, Tang TS, Cuba SL (2001). A comparison of blacks and whites seeking treatment for chronic pain. Clin J Pain.

[27] Meghani SH, Byun E, Gallagher RM (2012). Time to take stock: a meta-analysis and systematic review of analgesic treatment disparities for pain in the United States. Pain Med.

[28] (2011). Why not the best? Results from the national scorecard on U.S. health system performance, 2011. The Commonwealth Fund.

[29] Wasiak R, Pransky GS, Atlas SJ (2008). Who's in charge? Challenges in evaluating quality of primary care treatment for low back pain. J Eval Clin Pract.

[30] Olfson M, Wall M, Wang S, Crystal S, Blanco C (2018). Service use preceding opioid-related fatality. Am J Psychiatry.

[31] Balsa AI, McGuire TG (2003). Prejudice, clinical uncertainty and stereotyping as sources of health disparities. J Health Econ.

[32] Aronowitz SV, Mcdonald CC, Stevens RC, Richmond TS (2020). Mixed studies review of factors influencing receipt of pain treatment by injured black patients. J Adv Nurs.

[33] Larochelle MR, Liebschutz JM, Zhang F, Ross-Degnan D, Wharam JF (2016). Opioid prescribing after nonfatal overdose and association with repeated overdose: a cohort study. Ann Intern Med.

[34] (2021). CDC's efforts to prevent opioid overdoses and other opioid-related harms. Centers for Disease Control and Prevention.

[35] Bicego A, Monseur J, Collinet A, Donneau AF, Fontaine R, Libbrecht D, Malaise N, Nyssen AS, Raaf M, Rousseaux F, Salamun I, Staquet C, Teuwis S, Tomasella M, Faymonville ME, Vanhaudenhuyse A (2021). Complementary treatment comparison for chronic pain management: a randomized longitudinal study. PLoS One.

[36] Craner JR, Skipper RR, Gilliam WP, Morrison EJ, Sperry JA (2016). Patients' perceptions of a chronic pain rehabilitation program: changing the conversation. Curr Med Res Opin.

[37] Khoong EC, Butler BA, Mesina O, Su G, DeFries TB, Nijagal M, Lyles CR (2021). Patient interest in and barriers to telemedicine video visits in a multilingual urban safety-net system. J Am Med Inform Assoc.

[38] Schickedanz A, Huang D, Lopez A, Cheung E, Lyles CR, Bodenheimer T, Sarkar U (2013). Access, interest, and attitudes toward electronic communication for health care among patients in the medical safety net. J Gen Intern Med.

[39] Sarkar U, Lee JE, Nguyen KH, Lisker S, Lyles CR (2021). Barriers and facilitators to the implementation of virtual reality as a pain management modality in academic, community, and safety-net settings: qualitative analysis. J Med Internet Res.

[40] Baniasadi T, Ayyoubzadeh SM, Mohammadzadeh N (2020). Challenges and practical considerations in applying virtual reality in medical education and treatment. Oman Med J.

[41] Trost Z, Zielke M, Guck A, Nowlin L, Zakhidov D, France CR, Keefe F (2015). The promise and challenge of virtual gaming technologies for chronic pain: the case of graded exposure for low back pain. Pain Manag.

[42] (2013). Usability evaluation basics. Usability.gov.

[43] Sekhon M, Cartwright M, Francis JJ (2017). Acceptability of healthcare interventions: an overview of reviews and development of a theoretical framework. BMC Health Serv Res.

[44] Safety net. Agency for Healthcare Research and Quality.

[45] Lee H, Hill L, McConville S (2012). Access to the health care safety net in California. Public Policy Institute of California.

[46] Jha AK, DesRoches CM, Shields AE, Miralles PD, Zheng J, Rosenbaum S, Campbell EG (2009). Evidence of an emerging digital divide among hospitals that care for the poor. Health Aff (Millwood).

[47] Owens C, Farrand P, Darvill R, Emmens T, Hewis E, Aitken P (2011). Involving service users in intervention design: a participatory approach to developing a text-messaging intervention to reduce repetition of self-harm. Health Expect.

[48] McCurdie T, Taneva S, Casselman M, Yeung M, McDaniel C, Ho W, Cafazzo J (2012). mHealth consumer apps: the case for user-centered design. Biomed Instrum Technol.

[49] Moher D, Liberati A, Tetzlaff J, Altman DG, PRISMA Group (2009). Preferred Reporting Items for Systematic Reviews and Meta-Analyses: the PRISMA statement. Ann Intern Med.

[50] Mossey JM (2011). Defining racial and ethnic disparities in pain management. Clin Orthop Relat Res.

[51] Case A, Deaton A, Stone AA (2020). Decoding the mystery of American pain reveals a warning for the future. Proc Natl Acad Sci U S A.

[52] World Bank perspective on population aging. World Bank Group.

[53] (2022). Education worldwide—statistics & facts. Statista.

[54] Alegria M, Sud S, Steinberg BE, Gai N, Siddiqui A (2021). Reporting of participant race, sex, and socioeconomic status in randomized clinical trials in general medical journals, 2015 vs 2019. JAMA Netw Open.

[55] (2021). World Bank country and lending groups. World Bank.

[56] Arezki R, Yuting Fan R, Nguyen H (2019). Technology adoption and the middle‐income trap: lessons from the Middle East and East Asia. World Bank.

[57] (2013). Glossary. U.S. General Services Administration (GSA), Technology Transformation Service, Usability.gov.

[58] Fowler CA, Ballistrea LM, Mazzone KE, Martin AM, Kaplan H, Kip KE, Ralston K, Murphy JL, Winkler SL (2019). Virtual reality as a therapy adjunct for fear of movement in veterans with chronic pain: single-arm feasibility study. JMIR Form Res.

[59] Benham S, Kang M, Grampurohit N (2019). Immersive virtual reality for the management of pain in community-dwelling older adults. OTJR (Thorofare N J).

[60] Hennessy RW, Rumble D, Christian M, Brown DA, Trost Z (2020). A graded exposure, locomotion-enabled virtual reality app during walking and reaching for individuals with chronic low back pain: cohort gaming design. JMIR Serious Games.

[61] Garcia-Palacios A, Herrero R, Vizcaíno Y, Belmonte MA, Castilla D, Molinari G, Baños RM, Botella C (2015). Integrating virtual reality with activity management for the treatment of fibromyalgia: acceptability and preliminary efficacy. Clin J Pain.

[62] Stamm O, Dahms R, Müller-Werdan U (2020). Virtual reality in pain therapy: a requirements analysis for older adults with chronic back pain. J Neuroeng Rehabil.

[63] (2013). System Usability Scale (SUS). Usability.gov.

[64] Pourmand A, Davis S, Marchak A, Whiteside T, Sikka N (2018). Virtual reality as a clinical tool for pain management. Curr Pain Headache Rep.

[65] Gupta A, Scott K, Dukewich M (2018). Innovative technology using virtual reality in the treatment of pain: does it reduce pain via distraction, or is there more to it?. Pain Med.

[66] Mallari B, Spaeth EK, Goh H, Boyd BS (2019). Virtual reality as an analgesic for acute and chronic pain in adults: a systematic review and meta-analysis. J Pain Res.

[67] Khoja SS, Almeida GJ, Freburger JK (2020). Recommendation rates for physical therapy, lifestyle counseling, and pain medications for managing knee osteoarthritis in ambulatory care settings: a cross-sectional analysis of the national ambulatory care survey (2007-2015). Arthritis Care Res (Hoboken).

[68] Zheng P, Kao MC, Karayannis NV, Smuck M (2017). Stagnant physical therapy referral rates alongside rising opioid prescription rates in patients with low back pain in the United States 1997-2010. Spine (Phila Pa 1976).

[69] Goyal MK, Kuppermann N, Cleary SD, Teach SJ, Chamberlain JM (2015). Racial disparities in pain management of children with appendicitis in emergency departments. JAMA Pediatr.

[70] Indicator 17: high school status dropout rates. National Center for Education Statistics.

[71] Arevian AC, O'Hora J, Jones F, Mango J, Jones L, Williams PG, Booker-Vaughns J, Jones A, Pulido E, Banner-Jackson D, Wells KB (2018). Participatory technology development to enhance community resilience. Ethn Dis.

[72] Lyles CR, Aguilera A, Nguyen O, Sarkar U (2022). Bridging the digital health divide: how designers can create more inclusive digital health tools. California Health Care Foundation.

[73] Avila-Garcia P, Hernandez-Ramos R, Nouri SS, Cemballi A, Sarkar U, Lyles CR, Aguilera A (2019). Engaging users in the design of an mHealth, text message-based intervention to increase physical activity at a safety-net health care system. JAMIA Open.

[74] Nijagal MA, Patel D, Lyles C, Liao J, Chehab L, Williams S, Sammann A (2021). Using human centered design to identify opportunities for reducing inequities in perinatal care. BMC Health Serv Res.

[75] Lyles CR, Altschuler A, Chawla N, Kowalski C, McQuillan D, Bayliss E, Heisler M, Grant RW (2016). User-centered design of a tablet waiting room tool for complex patients to prioritize discussion topics for primary care visits. JMIR Mhealth Uhealth.

[76] Pathak LE, Aguilera A, Williams JJ, Lyles CR, Hernandez-Ramos R, Miramontes J, Cemballi AG, Figueroa CA (2021). Developing messaging content for a physical activity smartphone app tailored to low-income patients: user-centered design and crowdsourcing approach. JMIR Mhealth Uhealth.

[77] Sarkar U, Gourley GI, Lyles CR, Tieu L, Clarity C, Newmark L, Singh K, Bates DW (2016). Usability of commercially available mobile applications for diverse patients. J Gen Intern Med.

[78] Morales ME, Yong RJ (2021). Racial and ethnic disparities in the treatment of chronic pain. Pain Med.

[79] Rutledge T, Velez D, Depp C, McQuaid JR, Wong G, Jones RCW, Atkinson JH, Giap B, Quan A, Giap H (2019). A virtual reality intervention for the treatment of phantom limb pain: development and feasibility results. Pain Med.

[80] Thomas JS, France CR, Applegate ME, Leitkam ST, Walkowski S (2016). Feasibility and safety of a virtual reality dodgeball intervention for chronic low back pain: a randomized clinical trial. J Pain.

[81] Venuturupalli RS, Chu T, Vicari M, Kumar A, Fortune N, Spielberg B (2019). Virtual reality-based biofeedback and guided meditation in rheumatology: a pilot study. ACR Open Rheumatol.

