# Supplementary material for: Virtual Reality for Chronic Pain Management Among Historically Marginalized Populations: Systematic Review of Usability Studies
Source: J Med Internet Res. 2023 Jun 6;25:e40044. doi: 10.2196/40044 (PMC10282907; doi:10.2196/40044)
Supplement: Multimedia Appendix 1 [file jmir_v25i1e40044_app1.docx]

**Multimedia Appendix 1. Characteristics of full texts reviewed.**

| **Article** | **Exclude or Accept** | **Exclusion Reason** | **Study Design** | **Size (N)** | **Reported population demographics** | **Outcome type** |
| --- | --- | --- | --- | --- | --- | --- |
| Benham et al, 2019 | Accept |  | Observational (Mixed-methods pilot) | 12 | 92% Caucasian  67% female  Mean age= 70.2 years (± 3.6)** | Measure the effects of  VR on pain intensity and closely related factors. Track perceived experiences of participants to gauge applicability of VR among community-dwelling older adults. |
| Chau et al, 2020 | Exclude | Demographic criteria not met | Observational (Pilot) | 8 | Mean age= 45.4 years (± 20.4)  86% female | Explore the effects of VR on pain in patients with upper limb complex regional pain syndrome (CRPS). |
| Cole et al, 2009 | Exclude | Demographic criteria not met | Observational (Mixed-methods) | 14 | 71% male  Leg amputees, mean age= 49 years (± 16.0)  Arm amputees, mean age= 56 years (± 19.3) | Explore effects of VR for patients with phantom limb pain. Assess VR as an analgesic and patient’s sense of agency over virtual limb. |
| Darnall et al, 2020 | Exclude | Demographic criteria not met | Randomized Control Trial (Pilot) | 74 | VR group:  72% white, 14% African-American, 10% Hispanic/Latino, 3% Native American/Pacific Islander, 17% Missing  9% 25-34 years, 14% 35-44 years, 31% 45-54 years, 31% 55-64 years, 14% >65 years  74% male  48% high school graduate, 14% some college, 21% bachelor degree, 17% postgraduate, 17% missing  38% full time, 31% part time, 10% not working, 17% unable to work, 3% retired, 17% missing  52% married/civil union, 3% widowed, 17% divorced/ separated, 14% single/ cohabiting, 14% single, 17% missing  Audio group:  76% white, 8% African-American, 5% Asian, 5% Hispanic/Latino, 3% Native American/Pacific Islander, 3% multiracial/other, 5% missing  8% 25-34 years, 21% 35-44 years, 31% 45-54 years, 18% 55-64 years, 23% >65 years  67% male  8% some high school, 30% high school graduate, 5% some college, 35% bachelor degree, 22% postgraduate, 5% missing  43% full time employment, 19% part time, 8% not working, 27% unable to work, 3% retired, 5% missing  43% married/civil union, 3% widowed, 14% divorced /separated, 3% single/ cohabiting, 38% single, 5% missing | Evaluate feasibility and efficacy of VR program for chronic pain |
| Fowler et al, 2019 | Accept |  | Observational (Single-arm feasibility) | 16 | 81% male  50% white, 25% African-American or Black, 12% Hispanic or Latino, 13% other**  Mean age= 48.9 (± 11.6) | Assess effect and feasibility of VR usage on fear of movement, pain outcomes and patient functioning |
| Garcia-Palacios et al, 2015 | Accept |  | Randomized Control Trial | 61 | 100% Caucasian  100% female  Mean age= 50.5 years old (± 9.8)  21% less than elementary level of education, 36.1% elementary  level of education, 31.2% completed high school, 11.4% completed university**  79% married, 10% single, 10% divorced, 2% widow | Assess acceptability and efficacy of VR on activity level, pain, quality of life, and mood of patients with fibromyalgia |
| Garrett et al, 2017 | Exclude | Demographic criteria not met | Observational (Mixed-methods) | 10 | 89% female  Mean age= 51 (± 14.5) | Assess value of VR as an adjunctive therapy for chronic pain patients in their own homes |
| Garrett et al, 2020 | Exclude | Demographic criteria not met | Qualitative analysis of intervention arm of a randomized controlled trial | 12 | 50% male, 33 % female, 17% undisclosed  Age range: 37-73 years | Study patient experience with and perspectives on using VR for chronic cancer pain control |
| Hennessy et al, 2020 | Accept |  | Observational (Content validity and feasibility) | 12 | 100% Black**  67% female  Mean age= 54.3 (± 5.1) | Determine content validity and usability of VR for individuals with chronic low back pain (cLBP) |
| Matheve et al, 2020 | Exclude | Demographic criteria not met | Randomized Control Trial | 84 | VR Group:  64% female  Mean age= 42.1 (± 11.5)  Control Group:  64% female  Mean age= 44.2 (± 11.9) | Evaluate effect of VR on pain intensity and distraction from thoughts about pain. Assess patient motivation and perceived harmfulness of VR games. |
| Rutledge et al, 2019 | Exclude | Demographic criteria not met | Observational (Feasibility and acceptability) | 14 | Mean age= 63 (± 12.6)  93% male  64% Caucasian  79% > high school education  79% receiving care through VA hospital  64% married | Evaluate feasibility and acceptability of a novel VR intervention. Assess the benefits of VR treatment for reducing PLP intensity and phantom sensations. |
| Spyridonis et al, 2012 | Exclude | Demographic criteria not met | Observational (Usability evaluation) | 7 | None reported | Evaluate usability of Android application (PainDroid) with VR for the purpose of improving the management of pain for wheelchair users |
| Stamm et al, 2020 | Accept |  | Observational (Usability evaluation) | 15 | Mean age= 75.9 (± 6.9)** | Understand expectations, desires, preferences and barriers of VR pain therapy. Determine frameworks of therapy by physiotherapists and psychotherapists. |
| Thomas et al, 2016 | Exclude | Demographic criteria not met | Randomized Control Trial | 52 | VR group:  Mean age= 23.9 (±6.8)  46% female  80.8% white, 15% black, 4% more than one  4% Hispanic or Latino | Evaluate changes in lumbar spine flexion and expectations of pain and harm during VR intervention. Examine the feasibility and safety of a virtual dodgeball intervention for individuals with CLBP and pain-related fear. |
| Tong et al, 2016 | Exclude | Demographic criteria not met | Observational (Usability evaluation and efficacy) | 20 | 65% female | Evaluate usability and effect on patient pain/discomfort of two different VR displays |
| Venuturupalli et al, 2019 | Exclude | Demographic criteria not met | Observational (Mixed-methods pilot) | 17 | 88% Female  76 % Caucasian 12% Black, 6% Asian  Average age= 52.6 (±16.1) | Assess implementation of VR in a rheumatology clinic to administer guided meditation and biofeedback as a means of reducing chronic pain |
| Wiederhold et al, 2014 | Exclude | Demographic criteria not met | Observational (Usability evaluation) | 40 | Age range: 22–68 years | Evaluate usability of VR as a distraction technique for use as adjunctive therapy to treat chronic pain |

***Reported study demographic that met inclusion criteria*
